# A considerable proportion of CRF01_AE strains in China originated from circulating intrasubtype recombinant forms (CIRF)

**DOI:** 10.1186/s12879-015-1273-5

**Published:** 2015-11-16

**Authors:** Lei Jia, Tao Gui, Lin Li, Siyang Liu, Hanping Li, Zuoyi Bao, Xiaolin Wang, Daomin Zhuang, Tianyi Li, Jingwan Han, Yongjian Liu, Jingyun Li

**Affiliations:** Department of AIDS Research, State Key Laboratory of Pathogen and Biosecurity, Beijing Institute of Microbiology and Epidemiology, 20 Dongda Street, Fengtai District Beijing, 100071 China

**Keywords:** HIV-1, CRF01_AE, Intrasubtype recombination, Circulating intrasubtype recombinant forms, China

## Abstract

**Background:**

In this study, the prevalence of HIV-1 CRF01_AE intrasubtype recombinants in China is estimated and their contributions to the epidemic are explored.

**Methods:**

Available HIV-1 complete genomes of CRF01_AE were retrieved from the HIV database. The two alignments were evaluated with RDP3. Recombinants were defined as cases in which the recombination signal was supported by at least 3 methods with *P*-values of ≤0.05 after Bonferroni correction for multiple comparisons implemented in RDP3. Phylogenetic analysis was performed to further investigate the role of intrasubtype recombinants in epidemics.

**Results:**

Here, 124 out of the 339 sequences from around the world (36.6 %) showed significant evidence of recombination. Here, 84 of these recombinants were from China, accounting for 54.9 % of local total sequences (84 out of 153). The results indicated non-negligible levels of intrasubtype recombination. Subsequent phylogenetic analysis indicated that a considerable proportion of CRF01_AE strains in China originated from circulating intrasubtype recombinant forms. Three large, well-supported intrasubtype recombinants clusters were identified here. Through a survey of risk factors and sampling cities and provinces, cluster I and cluster II were found to be prevalent primarily among men who have sex with men in major northern cities. Cluster III was prevalent among heterosexuals and intravenous drug users in southern and southwestern provinces.

**Conclusions:**

The current work highlighted the remarkable prevalence of intrasubtype recombination within the CRF01_AE epidemic and emphasized the value of intrasubtype recombinants, which came to circulate in the same manner as intersubtype recombinants.

**Electronic supplementary material:**

The online version of this article (doi:10.1186/s12879-015-1273-5) contains supplementary material, which is available to authorized users.

## Background

Recombination is one of the two most important causes of the high prevalence of variation of human immunodeficiency virus type 1 (HIV-1) [1]. Analyses of recombination can reveal a great deal about evolution in general, much like analyses of nucleotide substitution can. The presence of multiple viral variants can produce HIV-1 recombinant strains that contain sequences either from different genetic subtypes (intersubtype recombination) or different viruses within the same subtype (intrasubtype recombination). Intersubtype recombination of HIV-1 occurs frequently and can produce many intersubtype recombinants, currently known to include 72 circulating recombinant forms (CRFs) of HIV-1 (http://www.hiv.lanl.gov/content/sequence/HIV/CRFs/CRFs.html) and numerous unique recombinant forms (URFs) of HIV-1. Intersubtype recombination is one major contributing cause of HIV-1 variability, and it facilitates the rapid generation of viral variants with high replicative capacity, drug resistance, or different expression of antigenic epitopes (summarized in [1, 2]). HIV-1 intersubtype recombination can be detected relatively easily [3]. However, HIV-1 intrasubtype recombination has been difficult to detect [4]. To date, most previous studies have focused on HIV-1 intersubtype recombination [5–7]. They have provided us with a very deep understanding of the evolutionary, clinical, and biological relevance, but only a few have addressed intrasubtype recombination in HIV-1. For example, two independent researches revealed frequent intrasubtype recombination among HIV-1 circulating in both South African and Tanzania [4, 8]. In this way, the HIV epidemic and other issues related to HIV-1 intrasubtype recombination have been overlooked. Historically, intersubtype recombinants of HIV-1 have been identified using subtype-specific reference sequences. However, due to the lack of such reference sequences, identification of HIV-1 intrasubtype recombinants has been limited to cases in which known sequences of transmitted multiple viral variants could serve as references for recombination analysis [8]. The introduction of recombination detection software RDP3 eliminates the need for reference sequences and allows us to estimate the prevalence of intrasubtype recombination of CRF01_AE in China [8, 9].

The HIV-1 CRF01_AE was initially named subtype E before it was found to be a recombinant virus [6, 10]. Phylogenetic analysis have indicated that CRF01_AE originated in central Africa in the 1970s and spread to Thailand in the 1980s through heterosexual transmission [10, 11]. Later it was confirmed as the first large-scale epidemic of an intersubtype recombinant around the world [6, 12]. In China, CRF01_AE strains were first identified in the early 1990s. They were found among persons at risk of sexual transmission and intravenous drug users (IDUs) in Yunnan and Guangxi, two provinces in southwestern China that border Myanmar and Vietnam [12–14]. According to the latest nationwide molecular epidemiological survey of newly reported cases in 2006, CRF01_AE strains quickly became the most widespread strains of HIV-1 according to geographic and risk group distributions, accounting for 28 % of nationwide HIV infections during the period [15]. Additionally, CRF01_AE have become the principal strain among men who have sex with men (MSM) in China [16, 17]. A recent study demonstrated that seven distinct phylogenetic clusters of CRF01_AE can be identified among CRF01_AE epidemics in China [18]. Molecular clock analysis indicated that multiple genetically distinct lineages were independently introduced to China from Southeast Asia during the mid-to-late 1990s and subsequently spread into different risk groups and geographic regions within China [18].

Based on the Los Alamos HIV database (http://www.hiv.lanl.gov/content/index) in which many CRF01_AE sequences have been collected, this study first addresses the prevalence of intrasubtype recombinants among near-full-length CRF01_AE genomes at the host population level in China and then evaluates their contributions to viral transmission and epidemics. The results showed significantly high prevalence of intrasubtype recombinants in circulation and indicated that a considerable proportion of CRF01_AE strains in China originated from circulating intrasubtype recombinant forms.

## Methods

### Selection of sequences

All available complete sequences were selected from the Los Alamos HIV-1 sequence database in September 2014 (one sequence/patient). A total of 346 CRF01_AE sequences were initially included in the alignment. All sequences were evaluated using a quality control tool (http://www.hiv.lanl.gov/content/sequence/QC/index.html). Seven sequences were found to be intersubtype recombinants and none were found to be hypermutated. Relevant sequences were removed, leaving 339 sequences for analysis. The country of origin and the sampling year(s) were summarized in Table 1. When downloading sequences, the *Align* checkbox was checked. After downloaded, minor manual adjustments were performed. The alignment was defined as dataset 1. Then 153 sequences from China were copied from dataset 1 and the new alignment was defined as dataset 2. Gap squeezing (i.e., columns that were entirely gaps were deleted) was performed on both datasets. Both datasets are available upon request.

**Table 1:** Geographic origin of sequences and sampling year

Country	No. of sequences	Sampling year(s)
AFGHANISTAN (AF)	1	2007
CENTRAL AFRICAN REPUBLIC (CF)	1	1990
IRAN (IR)	1	2010
CHINA (CN)	153	1997–2011
HONG KONG (HK)	1	2004
INDONESIA (ID)	1	1993
JAPAN (JP)	11 (8 sequences have no sampling year)	1993–2011
THAILAND (TH)	133 (1 sequences have no sampling year)	1990–2009
UNITED STATES (US)	4	1998–2005
VIETNAM (VN)	33	1997–1998
Total	339	1990–2011

### Identification of recombination

RDP3, a recombination analysis tool for statistical identification and characterization of recombination events in nucleotide sequences, was used to perform the analysis [8, 9]. RDP3 simultaneously uses a range of non-parametric recombination detection methods: RDP, Geneconv [19], Bootscan [20, 21], Maxchi [22, 23], Chimaera [22], SiScan [24], and 3Seq [25]. RDP3 treats every sequence within the analyzed sequence alignment as a potential recombinant. It systematically screens sequence triplets or quartets to identify a recombinant sequence and two specific sequences that could serve as parents. At the same time, RDP3 performs a statistical evaluation of recombination signals [8, 9]. This approach eliminates the need for reference sequences [8, 9, 26]. The main strength of RDP3 is that it simultaneously uses several different methods to both detect and characterize the recombination events within a sequence alignment without any prior user indication of a non-recombinant set of reference sequences [8, 9]. The sequences are set to *linear*. The highest acceptable *P*-value is set to 0.05. Within bootscan analysis, step-size was set to 50 and bootstrap replicates were set to 80 due to file size restriction. The other parameters are default RDP3 settings. An HIV-1 sequence was considered to be recombinant when the recombination signal was supported by at least 3 methods with *P*-values of ≤0.05 after Bonferroni correction for multiple comparisons implemented in RDP3 [8, 9, 27]. The breakpoint positions predicted were manually checked using recombination signal analysis implemented in RDP3. Although “list events detected by 3 methods” was selected, only 9 out of 22 events were supported by only 3 methods in these results. All others were supported by >3 methods. The subsequently identified cluster I (corresponding to event 19) and cluster II (corresponding to event 20) were both supported by 4 methods. Cluster III (corresponding to event 22) was supported by 5 methods.

### Phylogenetic analysis

jModelTest v0.1.1 was used to find the best-fitting model of nucleotide substitution for the dataset 2 [28]. The general time reversible (GTR) model with g-distributed (G) among-site rate heterogeneity and a proportion of invariant sites (I) (the GTR + I + G model) were chosen as the most appropriate model on the basis of the standard Akaike information criterion (AIC) and Bayesian information criterion (BIC). PhyML 3.0 was used to estimate a maximum likelihood phylogenetic tree for near full-length genome (NFLG) sequences in China using the GTR + I + G nucleotide substitution model [29]. Tree topologies were searched heuristically using the subtree pruning and regrafting procedure (SPR). The confidence of each node in phylogenetic trees was determined using the fast-likelihood-based method of aLRT SH-like. The final maximum likelihood tree was visualized using Mega5 [30]. The topology of the tree was further confirmed by another round of phylogenetic analysis using a Beast tool based on *Bayesian inference* (data not shown).

## Results

### Prevalence of intrasubtype recombination of CRF01_AE among populations in China

The alignment of local NFLG sequences from 153 subjects from China infected with HIV-1 CRF01_AE (dataset 2) were first analyzed for evidence of intrasubtype recombination. The purpose of this round of intrasubtype recombination analysis under the background of mainland China was to compare its results to those of the subsequent rounds of intrasubtype recombination analysis of CRF01_AE sequences in mainland China under the background of all over the world, i.e., the background of 339 sequences.

**Fig. 1:**
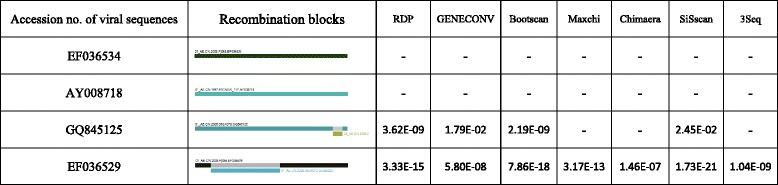
HIV-1 CRF01_AE intrasubtype recombination analysis by RDP3. RDP3 methods with supporting *P*-values and recombination patterns are listed for 4 examples. Colored rectangles indicate sequence segments and the small rectangles indicate the putative recombinant regions. The absence of *P*-value indicates that no recombination event was detected using the method specified

Recombination analysis was performed using seven methods implemented in RDP3. The results indicated a significant level of recombination. 16 out of 153 (10.5 %) sequences are intrasubtype recombinant viruses (Table 2), corresponding to 8 recombination events. Figure 1 shows an example of recombination analysis results for 4 strains. The first two (accession numbers: EF036534 and AY008718) did not show any intrasubtype recombination evidence. The third stain (accession number: GQ845125) has a short segment insertion, with 4 methods supported. By contrast, the fourth strain (accession number: EF036529) showed a large segment insertion with 7 methods supported.

**Table 2:** Comparison of results of Chinese CRF01_AE strains intrasubtype analysis between different background areas (mainland China vs all over the world)

Background area	No. of sequences from China	Sequences inferred as recombinant	Rate
Mainland China^a^	153	16	10.5 %
Worldwide^b^	153	84	54.9 %

^a^Performance of intrasubtype recombination analysis of CRF01_AE sequences in mainland China under the background of mainland China, i.e., the background of 153 sequences

^b^Performance of intrasubtype recombination analysis of CRF01_AE sequences in mainland China under the background of all over the world, i.e., the background of 339 sequences

Further analysis was performed on the alignment of 339 CRF01_AE sequences around the world (dataset 1). Results suggested that there are 124 recombinant sequences, accounting for 36.6 % of the total. The putative recombinant sequences corresponding to specific recombination event are listed in Additional file 1. The recombinants were found to be associated with 22 separate recombination events. The most shocking thing was that 84 out of the 124 were from China. The results under the background of mainland China indicated that only 16 were recombinant, but when evaluated against the background of the whole world, many more Chinese sequences were proven to be recombinants (84 versus16). The updated prevalence was as high as 54.9 %, reflecting a significantly high prevalence of intrasubtype recombinants in circulation (Table 2). The apparent discrepancy between the 2 analyses indicated that, when placed in a much wider scope, more strains were connected with either minor or major parents, and therefore much more accurate indication of the amount of intrasubtype recombination can be found. Such results also indicate how background areas might best be selected when performing similar analyses.

### Phylogenetic and recombination analysis indicated that considerable CRF01_AE strains in China originated from circulating intrasubtype recombinant forms

Among these 124 recombinants, 1 was from Afghanistan, and all the others were from China (84 recombinants), Thailand (14 recombinants), or Vietnam (25 recombinants), which are geographically close. Such clustering in geographic distribution and the fact that distinct CRF01_AE lineages were independently introduced to China from Southeast Asia suggest that these intrasubtype recombinants very likely originated within this region and that there have probably been epidemics caused by intrasubtype recombinants from the same recombination event.

**Fig. 2:**
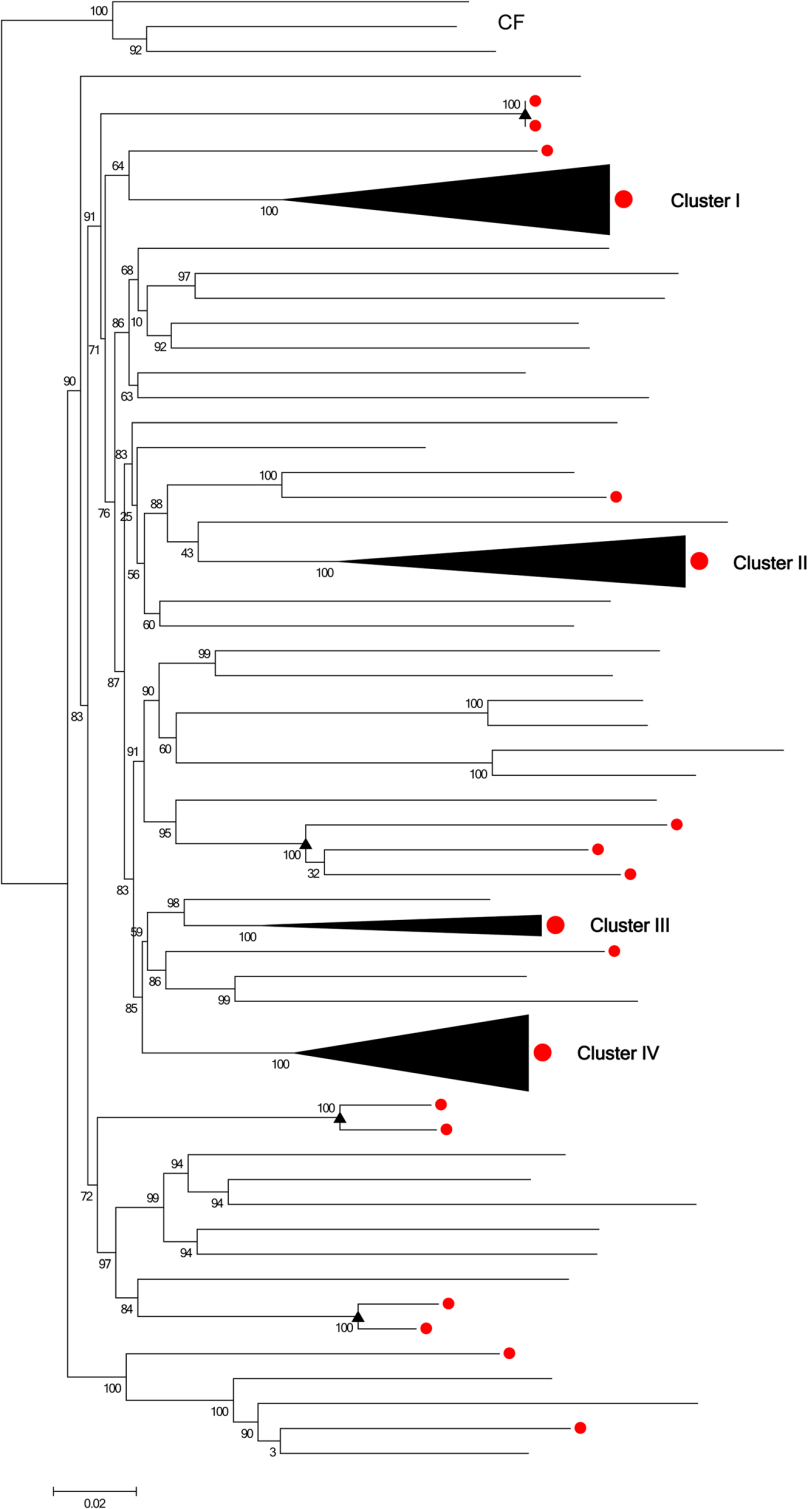
Recombinants located in the phylogenetic tree of NFLG HIV-1 CRF01_AE strains from China. Near-full-length genome (NFLG) sequences from China (*n* = 153) were analyzed in a maximum likelihood phylogenic tree. The tree was constructed using PhyML. SH values of all relevant nodes are indicated. SH support values of ≥90 % were here considered significant. A total of 84 CRF01_AE strains of 153 were found to be intrasubtype recombinants. All 84 sequences showing significant evidence (recombinants) are here labeled with red dots. Three large (≥10 sequences), well-supported (SH values were 100 %) and distinct clusters of CRF01_AE strains being almost entirely composed of putative recombinants were indicated from clusters I–III. Much smaller recombinant clusters including only 2 or 3 sequences are indicated by triangles. Cluster IV (SH value was 100 %) represents a nonrecombinant cluster. The outgroup is indicated with CF

**Fig. 3:**
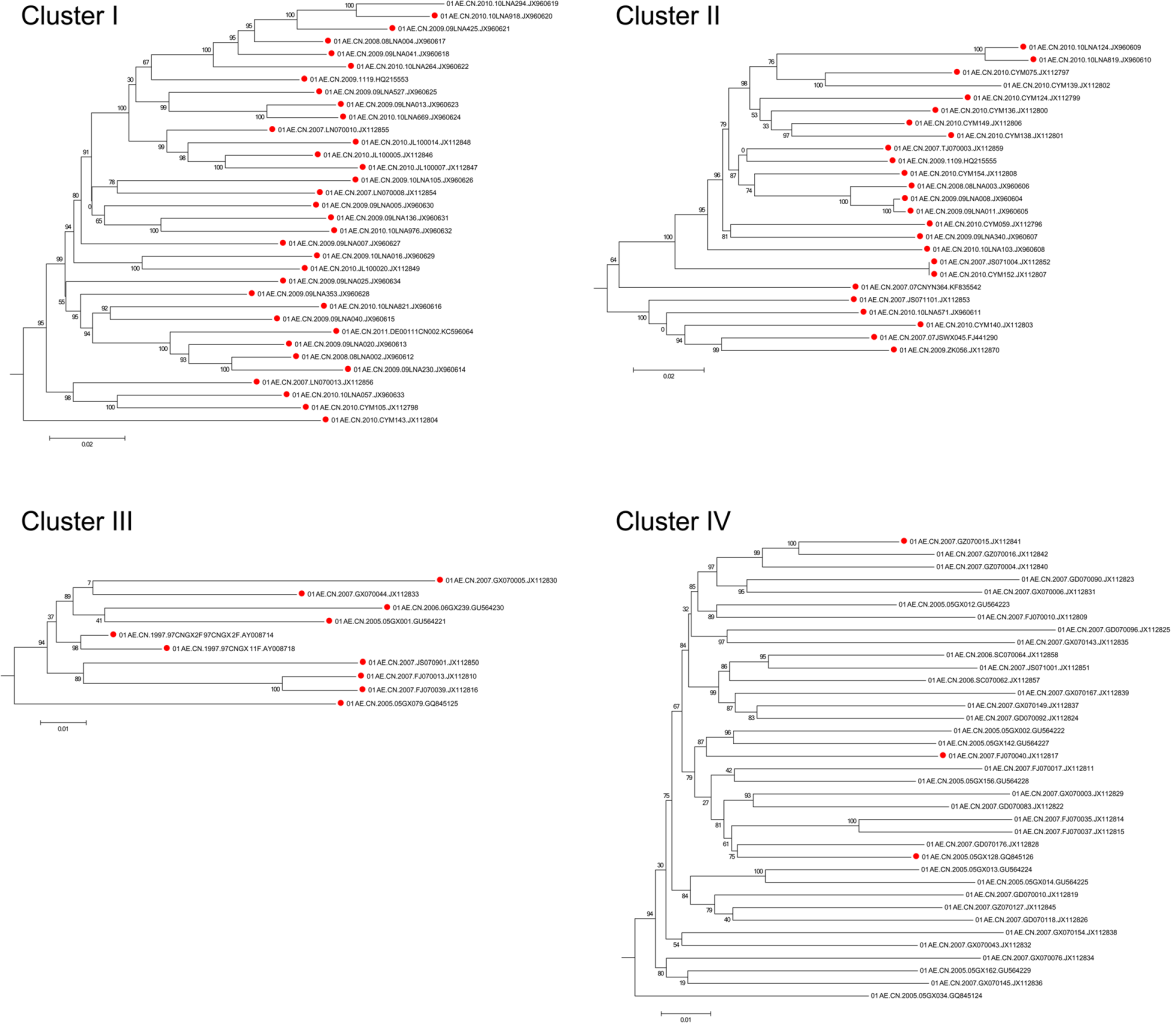
Details of the 4 clusters revealed from the phylogenetic tree of NFLG HIV-1 CRF01_AE strains from China. The trees were constructed using PhyML. SH values of all relevant nodes are indicated. SH support values of ≥90 % were here considered significant. Sequences showing significant evidence (recombinants) are labeled with red dots

In order to test this hypothesis, phylogenetic analysis was performed on dataset 2 which is based on mainland China. An additional 3 sequences from Central African Republic served as an outgroup. All 84 recombinants are indicated in the phylogenetic tree with red solid circles (Figs. 2 and 3). In the maximum-likelihood phylogeny of the near-full-length sequences, there existed several significant and well supported clusters. The present work focused on four large (≥10 sequences), well-supported (SH values were 100 %), and distinct clusters of CRF01_AE strains including clusters I, II, III, and IV (Figs. 2 and 3). These four clusters showed a surprising phenomenon: most recombinants were not evenly distributed among the ML tree but rather concentrated in the first three clusters, showing significant recombinant clustering (Figs. 2 and 3). In cluster I, 33 out of the 34 sequences were recombinants. In cluster II, 24 out of the 25 sequences were recombinants (The two sequences in clusters I and II did not show recombinant signals in RDP3 (accession numbers JX960619 and JX112802). This may be due to their poor sequence quality or the sensibility of RDP software.). In cluster III, all 10 sequences were recombinants. In contrast, only 3 of 37 are recombinants in cluster IV. Further identification showed that the first 2 recombinant clusters corresponded to specific recombination events. For example, the well-supported cluster I corresponded to recombination event number 19, the breakpoint positions of which were HXB2 nt 7240–7617 (7982–8523 in alignment) (Additional file 1 and Additional file 2). Cluster II corresponded to recombination event number 20, the breakpoint positions of which were HXB2 nt 7312–7610 (8091–8497 in alignment). All 2 recombination events were strongly evidenced (event 19 and 20 with 4 methods supported). Take cluster 1 for example, these results indicated the following: (1) The 33 recombinant sequences in this cluster were closely related to each other and shared a common ancestor; (2) all 33 recombinants in the cluster shared a common recombination event (recombination event number 19); (3) all these recombinants exhibited the same recombination pattern. In addition, another very important indication was that all 33 recombinant variants in cluster I shared the same parents. However, due to independent and rapid evolution of cluster I variants and considerable genomic similarity between sequences of the same subtype, RDP3 can only identify possible parents and not necessarily the real ones (Additional file 3) even after recombination signals have been confirmedly acquired. Even so, the first three pieces of evidence described above are sufficient to conclude that cluster I variants originated from an intrasubtype recombinant virus (established epidemiologically important founder strains). That is to say, new viruses have emerged and circulated after intrasubtype recombination events in the same manner as the CRFs that occur after intersubtype recombination events. These strains are described here as circulating intrasubtype recombinant forms. The explanations for the other clusters (II) are the same.

CRF01_AE was confirmed as the first large-scale epidemic of an intersubtype recombinant HIV-1 strain in the world [6, 12]. The current analysis revealed that intrasubtype recombination of CRF01_AE also contributed greatly to the circulating among HIV-1 population in China. In addition to these 3 big recombinant clusters, there were also exits several much smaller recombinant clusters including only 2 or 3 recombinant sequences (indicated by triangles). Unlike large clusters, they underwent intrasubtype recombination but did not seem to have caused the transmission rate to increase.

In cluster IV, only 3 of 37 sequences were detected as recombinants (accession number: JX112841, JX112817, and GQ845126). These three sequences corresponded to different recombinant events. The first 2 recombinants were associated with recombinant event 6 and the third with recombinant event 17. This is markedly different from recombinant clusters I and II, which were traced to a specific intrasubtype recombinant event, respectively. This case indicated that there first was an extensive epidemic caused by the pure subtype. The scattered intrasubtype recombinants were generated during this period of widespread circulation. The current work revealed there to have almost the same characteristics and rules within intrasubtype recombination and within intersubtype recombination. Take these three intrasubtype recombinants for example, they were the same as the unique recombinant forms (URF), in intersubtype recombination.

It is important to point out, besides the common Recombinant Event 22 (with 5 methods supported, the breakpoint positions of which were HXB2 nt 7156–7549), 5 sequences in cluster III also contained secondary recombinations: GU564230, GU564221, AY008714, and JX112810 are also involved in Recombinant Event 11. GQ845125 is also involved in Recombinant Event 10. In addition, the non-clustered, unique putative recombinant sequence of EF036533 was found to contain three types of segment insertion, so it has three Recombination Event Numbers, including Event 1, Event 2, and Event 20. These phenomena suggest that these 6 strains have undergone multiple recombination events.

Through a survey on the risk factor and sampling cities and provinces, the current cluster I and the current cluster II of intrasubtype recombinants were both found primarily among MSM in major northern cities while the current cluster III was prevalent among heterosexuals and IDUs in southern and southwestern provinces (Additional file 4).

## Discussion

Due to the very short distances between strains, intrasubtype recombination is much more difficult to detect with sequence analysis techniques than intersubtype recombination is. Additionally, recombination analysis also always requires reference sequences. However, such sequences are difficult to find for intrasubtype recombination. Therefore, unlike intersubtype recombination, which has attracted a great deal of attention, research on intrasubtype recombination is relatively sparse. RDP3 treats each sequence within the analyzed sequence alignment as a potential recombinant. Then it systematically screens sequence triplets or quartets to identify a recombinant and two specific sequences that could serve as parents during statistical evaluation of recombination signals [8, 9]. This approach can eliminate the need for reference sequences. Moreover, RDP3 also uses a range of different recombination detection methods to both detect and characterize the recombination events that are evident within the sequence alignment [8, 9]. This can increase the sensitivity of the detection process considerably. This produces more accurate results.

Results in this study first demonstrated that intrasubtype recombination can be detected and is frequent among near-full-length HIV-1 subtype CRF01_AE genomes. In the CRF01_AE epidemic in China, as many as 54.9 % of local total sequences (84 out of 153) are indicated as intrasubtype recombinants. This indicates that the epidemic in China merits further study. The present work demonstrated for the first time that, like intersubtype recombinants that have caused epidemics, such as CRF02_AG, CRF07_BC, CRF08_BC, and BF [31–34], intrasubtype recombinants from the same recombination event can also lead to the prevalence of HIV-1 and AIDS. As shown in Fig. 2 and Additional file 4, intrasubtype recombinants from cluster I and cluster II were found primarily among MSM in major northern cities. Intrasubtype recombinants within cluster III were prevalent among heterosexuals and IDUs in southern and southwestern provinces. Such strains are described in the present work as circulating intrasubtype recombinant forms, echoing circulating recombinant forms. This second term is used to describe intersubtype recombinants that have caused epidemic.

Several previous studies of intrasubtype recombination have concentrated on individual patients [35–37]. Others have focused on populations but within a single geographic location [4]. This work is the first to estimate intrasubtype recombination of viral sequences of CRF01_AE at the host population level using NFLG sequences around the world. As the data show, when placed in a much wider scope, the major and/or minor parents of more strains can be found, and this provides a much more accurate amount of intrasubtype recombination.

The prevalence of intrasubtype recombination provides important evidence of frequent intrasubtype dual infections at the level of populations because for detectable recombination, a host must be first infected by two different viral strains and then these strains must infect a common cell, become copackaged, and generate an infectious new progeny.

There still are several limitations of the work. First, the study was based on sequences that have been submitted to HIV database (the most recent sequences were submitted in 2011). In this way, the results may not reflect the most recent state of HIV infection. Second, although RDP3 can provide a much better performance for intrasubtype recombination detection via simultaneously using seven methods, there still exists limitation in sensitivity due to the very short genetic distances within sequences from the same subtype. With the development of high-throughput sequencing and the availability of more advanced recombination detection software, more optimized analysis may be possible. This would provide even more accurate information regarding intrasubtype recombination.

## Conclusions

In summary, the current work highlighted the remarkable prevalence of intrasubtype recombination within the CRF01_AE epidemic and emphasized the value of intrasubtype recombinants, which came to circulate in the same manner as intersubtype recombinants. All these results indicated that much more attention should be paid to intrasubtype recombination.

## Additional files

Additional file 1:
**The putative recombinant sequences of each recombination event.** (DOCX 21 kb) Additional file 2:
**The breakpoint positions of the recombinants within the 4 specified clusters relative to HXB2 (Genbank accession no. K03455).** (DOCX 16 kb) Additional file 3:
**The potential parents of recombinants within cluster I corresponding to recombination event 19.** (DOCX 16 kb) Additional file 4:
**Risk factors and sampling cities and provinces of sequences in clusters I–III.** (XLSX 12 kb)
