# Supplementary material for: A considerable proportion of CRF01_AE strains in China originated from circulating intrasubtype recombinant forms (CIRF)
Source: BMC Infect Dis. 2015 Nov 16;15:528. doi: 10.1186/s12879-015-1273-5 (PMC4647519; doi:10.1186/s12879-015-1273-5)
Supplement: Additional file 1: — The putative recombinant sequences of each recombination event. (DOCX 21 kb) [file 12879_2015_1273_MOESM1_ESM.docx]

Additional file 1. The putative recombinant sequences of each recombination event.

| Recombination Event Number | Putative Recombinant Sequence(s) |
| --- | --- |
| 1 | 01_AE.CN.2006.Fj064.EF036533 |
| 2 | 01_AE.CN.2006.Fj064.EF036533 |
| 3 | 01_AE.VN.1997.97VNHCM343.FJ185243 |
| 4 | 01_AE.VN.1997.97VNAG212.FJ185252 |
| 5 | 01_AE.CN.2005.Fj056.EF036529 |
| 6 | 01_AE.CN.2007.GZ070015.JX112841 |
|  | 01_AE.CN.2007.FJ070040.JX112817 |
| 7 | 01_AE.VN.1997.97VNAG201.FJ185245 |
| 8 | 01_AE.VN.1997.97VNAG214.FJ185253 |
|  | 01_AE.VN.1997.97VNHCM343.FJ185243 |
|  | 01_AE.VN.1997.97VNAG212.FJ185252 |
| 9 | 01_AE.VN.1997.97VNAG212.FJ185252 |
| 10 | 01_AE.CN.2005.05GX079.GQ845125 |
| 11 | 01_AE.VN.1997.97VNAG221.FJ185257 |
|  | 01_AE.CN.1997.97CNGX2F_97CNGX_2F.AY008714 |
|  | 01_AE.VN.1998.98VNBG4.FJ185228 |
|  | 01_AE.VN.1998.98VNBG6.FJ185230 |
|  | 01_AE.VN.1998.98VNBG7.FJ185231 |
|  | 01_AE.VN.1998.98VNHD9.FJ185232 |
|  | 01_AE.VN.1998.98VNHD10.FJ185233 |
|  | 01_AE.VN.1998.98VNHD11.FJ185234 |
|  | 01_AE.VN.1998.98VNND17.FJ185236 |
|  | 01_AE.VN.1997.97VNHCM303.FJ185239 |
|  | 01_AE.VN.1997.97VNHCM310.FJ185240 |
|  | 01_AE.VN.1997.97VNAG201.FJ185245 |
|  | 01_AE.VN.1997.97VNAG220.FJ185256 |
|  | 01_AE.VN.1997.97VNHCM306.FJ185259 |
|  | 01_AE.VN.1997.97VNHCM309.FJ185260 |
|  | 01_AE.CN.2005.05GX001.GU564221 |
|  | 01_AE.CN.2006.06GX239.GU564230 |
|  | 01_AE.CN.2007.FJ070013.JX112810 |
| 12 | 01_AE.VN.1997.97VNHCM314.FJ185241 |
|  | 01_AE.VN.1997.97VNHCM345.FJ185244 |
| 13 | 01_AE.VN.1997.97VNAG214.FJ185253 |
| 14 | 01_AE.CN.2005.FJ051.DQ859178 |
|  | 01_AE.CN.2002.YN0225.JX112862 |
|  | 01_AE.CN.2002.YN0235.JX112865 |
|  | 01_AE.CN.2009.ZK052.JX112869 |
| 15 | 01_AE.TH.1993.93TH062.AB220947 |
| 16 | 01_AE.CN.2007.07CNYN326.KF835513 |
|  | 01_AE.CN.2005.Fj052.EF036528 |
|  | 01_AE.AF.2007.569M.GQ477441 |
|  | 01_AE.TH.2004.04TH427990.JN248327 |
|  | 01_AE.CN.2007.GD070120.JX112827 |
|  | 01_AE.CN.2007.GZ070123.JX112843 |
|  | 01_AE.TH.2006.AA006a02.JX446736 |
|  | 01_AE.TH.2006.AA056a_WG2.JX447315 |
|  | 01_AE.TH.2006.AA088a_wg11.JX447709 |
| 17 | 01_AE.VN.1998.98VNBG5.FJ185229 |
|  | 01_AE.TH.2001.OUR788I.AY358068 |
|  | 01_AE.TH.1998.98TH_R1166.AY945728 |
|  | 01_AE.VN.1998.98VNBG7.FJ185231 |
|  | 01_AE.VN.1998.98VNND15.FJ185235 |
|  | 01_AE.VN.1997.97VNHCM301.FJ185237 |
|  | 01_AE.VN.1997.97VNHCM302.FJ185238 |
|  | 01_AE.VN.1997.97VNHCM310.FJ185240 |
|  | 01_AE.VN.1997.97VNHCM319.FJ185242 |
|  | 01_AE.VN.1997.97VNHCM309.FJ185260 |
|  | 01_AE.CN.2005.05GX128.GQ845126 |
|  | 01_AE.CN.2002.YN0229.JX112863 |
|  | 01_AE.CN.2002.YN0232.JX112864 |
|  | 01_AE.TH.2007.AA030a08.JX447054 |
| 18 | 01_AE.CN.2007.GZ070126.JX112844 |
|  | 01_AE.TH.2001.OUR788I.AY358068 |
| 19 | 01_AE.CN.2011.DE00111CN002.KC596064 |
|  | 01_AE.CN.2009.1119.HQ215553 |
|  | 01_AE.CN.2010.CYM105.JX112798 |
|  | 01_AE.CN.2010.CYM143.JX112804 |
|  | 01_AE.CN.2010.JL100005.JX112846 |
|  | 01_AE.CN.2010.JL100007.JX112847 |
|  | 01_AE.CN.2010.JL100014.JX112848 |
|  | 01_AE.CN.2010.JL100020.JX112849 |
|  | 01_AE.CN.2007.LN070008.JX112854 |
|  | 01_AE.CN.2007.LN070010.JX112855 |
|  | 01_AE.CN.2007.LN070013.JX112856 |
|  | 01_AE.TH.2005.AA033a_wg1.JX447077 |
|  | 01_AE.CN.2008.08LNA002.JX960612 |
|  | 01_AE.CN.2009.09LNA020.JX960613 |
|  | 01_AE.CN.2009.09LNA230.JX960614 |
|  | 01_AE.CN.2009.09LNA040.JX960615 |
|  | 01_AE.CN.2010.10LNA821.JX960616 |
|  | 01_AE.CN.2008.08LNA004.JX960617 |
|  | 01_AE.CN.2009.09LNA041.JX960618 |
|  | 01_AE.CN.2010.10LNA918.JX960620 |
|  | 01_AE.CN.2009.09LNA425.JX960621 |
|  | 01_AE.CN.2010.10LNA264.JX960622 |
|  | 01_AE.CN.2009.09LNA013.JX960623 |
|  | 01_AE.CN.2010.10LNA669.JX960624 |
|  | 01_AE.CN.2009.09LNA527.JX960625 |
|  | 01_AE.CN.2009.10LNA105.JX960626 |
|  | 01_AE.CN.2009.09LNA007.JX960627 |
|  | 01_AE.CN.2009.09LNA353.JX960628 |
|  | 01_AE.CN.2009.10LNA016.JX960629 |
|  | 01_AE.CN.2009.09LNA005.JX960630 |
|  | 01_AE.CN.2009.09LNA136.JX960631 |
|  | 01_AE.CN.2010.10LNA976.JX960632 |
|  | 01_AE.CN.2010.10LNA057.JX960633 |
|  | 01_AE.CN.2009.09LNA025.JX960634 |
| 20 | 01_AE.CN.2007.07JSWX045.FJ441290 |
|  | 01_AE.TH.2002.OUR737I.AY358037 |
|  | 01_AE.TH.2002.OUR769I.AY358062 |
|  | 01_AE.CN.2006.Fj064.EF036533 |
|  | 01_AE.CN.2009.1109.HQ215555 |
|  | 01_AE.CN.2010.CYM059.JX112796 |
|  | 01_AE.CN.2010.CYM075.JX112797 |
|  | 01_AE.CN.2010.CYM124.JX112799 |
|  | 01_AE.CN.2010.CYM136.JX112800 |
|  | 01_AE.CN.2010.CYM138.JX112801 |
|  | 01_AE.CN.2010.CYM140.JX112803 |
|  | 01_AE.CN.2010.CYM149.JX112806 |
|  | 01_AE.CN.2010.CYM152.JX112807 |
|  | 01_AE.CN.2010.CYM154.JX112808 |
|  | 01_AE.CN.2007.FJ070043.JX112818 |
|  | 01_AE.CN.2007.JS071004.JX112852 |
|  | 01_AE.CN.2007.JS071101.JX112853 |
|  | 01_AE.CN.2007.TJ070003.JX112859 |
|  | 01_AE.CN.2009.ZK056.JX112870 |
|  | 01_AE.TH.2008.AA067A_WG17.JX447454 |
|  | 01_AE.CN.2009.09LNA008.JX960604 |
|  | 01_AE.CN.2009.09LNA011.JX960605 |
|  | 01_AE.CN.2008.08LNA003.JX960606 |
|  | 01_AE.CN.2009.09LNA340.JX960607 |
|  | 01_AE.CN.2010.10LNA103.JX960608 |
|  | 01_AE.CN.2010.10LNA124.JX960609 |
|  | 01_AE.CN.2010.10LNA819.JX960610 |
|  | 01_AE.CN.2010.10LNA571.JX960611 |
|  | 01_AE.CN.2007.07CNYN364.KF835542 |
| 21 | 01_AE.TH.2000.OUR721I.AY358067 |
| 22 | 01_AE.VN.1997.97VNHCM310.FJ185240 |
|  | 01_AE.CN.1997.97CNGX2F_97CNGX_2F.AY008714 |
|  | 01_AE.CN.1997.97CNGX_11F.AY008718 |
|  | 01_AE.VN.1998.98VNBG4.FJ185228 |
|  | 01_AE.VN.1998.98VNBG5.FJ185229 |
|  | 01_AE.VN.1998.98VNBG6.FJ185230 |
|  | 01_AE.VN.1998.98VNHD9.FJ185232 |
|  | 01_AE.VN.1998.98VNHD10.FJ185233 |
|  | 01_AE.VN.1998.98VNHD11.FJ185234 |
|  | 01_AE.VN.1998.98VNND15.FJ185235 |
|  | 01_AE.VN.1997.97VNHCM302.FJ185238 |
|  | 01_AE.VN.1997.97VNAG201.FJ185245 |
|  | 01_AE.VN.1997.97VNAG207.FJ185249 |
|  | 01_AE.VN.1997.97VNAG220.FJ185256 |
|  | 01_AE.VN.1997.97VNAG221.FJ185257 |
|  | 01_AE.VN.1997.97VNHCM306.FJ185259 |
|  | 01_AE.VN.1997.97VNHCM309.FJ185260 |
|  | 01_AE.CN.2005.05GX079.GQ845125 |
|  | 01_AE.CN.2005.05GX001.GU564221 |
|  | 01_AE.CN.2006.06GX239.GU564230 |
|  | 01_AE.CN.2007.FJ070013.JX112810 |
|  | 01_AE.CN.2007.FJ070039.JX112816 |
|  | 01_AE.CN.2007.GX070005.JX112830 |
|  | 01_AE.CN.2007.GX070044.JX112833 |
|  | 01_AE.CN.2007.JS070901.JX112850 |
|  | 01_AE.TH.2006.AA017a_wg1.JX446899 |

A recombination event number indicates a specified type of segment exchange.
