# Supplementary material for: A considerable proportion of CRF01_AE strains in China originated from circulating intrasubtype recombinant forms (CIRF)
Source: BMC Infect Dis. 2015 Nov 16;15:528. doi: 10.1186/s12879-015-1273-5 (PMC4647519; doi:10.1186/s12879-015-1273-5)
Supplement: Additional file 2: — The breakpoint positions of the recombinants within the 4 specified clusters relative to HXB2 (Genbank accession no. K03455). (DOCX 16 kb) [file 12879_2015_1273_MOESM2_ESM.docx]

Additional file 2. The breakpoint positions of the recombinants within the 4 specified clusters relative to HXB2 (Genbank accession no. K03455).

| Cluster | Begin-end |
| --- | --- |
| I | 7240-7617 |
| II | 7312-7610 |
| III (recombinant event 22) | 7156-7549 |
| IV(accession no. JX112841 and JX112817) | 264* - 874 |
| IV(accession no. GQ845126) | 6632-6682 |

* The actual breakpoint position is undetermined.
