# Supplementary material for: A considerable proportion of CRF01_AE strains in China originated from circulating intrasubtype recombinant forms (CIRF)
Source: BMC Infect Dis. 2015 Nov 16;15:528. doi: 10.1186/s12879-015-1273-5 (PMC4647519; doi:10.1186/s12879-015-1273-5)
Supplement: Additional file 3: — The potential parents of recombinants within cluster I corresponding to recombination event 19. (DOCX 16 kb) [file 12879_2015_1273_MOESM3_ESM.docx]

Additional file 3. The potential parents of recombinants within cluster I corresponding to recombination event 19 .

| Recombination Event Number | Minor Parental Sequence(s) | Major Parental Sequence(s) |
| --- | --- | --- |
| 19 | 01_AE.CN.2007.07CNYN364.KF835542  01_AE.CN.2010.CYM075.JX112797  01_AE.CN.2010.CYM149.JX112806  01_AE.CN.2007.JS071101.JX112853  01_AE.CN.2009.ZK056.JX112870 | Unknown*^a^*(01_AE.VN.1997.97VNHCM301.FJ185237)  Unknown*^a^*(01_AE.TH.1993.93TH253.U51189) |

*^a^* The sequence listed as unknown was used to infer the existence of a missing parental sequence.
